# Fractures in elderly mice demonstrate delayed ossification of the soft callus: a cellular and radiographic study

**DOI:** 10.1007/s00590-022-03235-w

**Published:** 2022-03-03

**Authors:** N. D. Clement, M. S. Gaston, A. H. Simpson

**Affiliations:** Department of Orthopaedics and Trauma, University of Edinburgh, Little France, Edinburgh, EH16 4SA UK

**Keywords:** Fracture, Healing, Aging, Callus, Delay, Rodent

## Abstract

**Objectives:**

The aim of this study was to assess the cellular age-related changes in fracture repair and relate these to the observed radiographic assessments at differing time points.

**Methods:**

Transverse traumatic tibial diaphyseal fractures were created in 12–14 weeks old (young *n* = 16) and 18 months old (elderly *n* = 20) in Balb/C wild mice. Fracture calluses were harvested at five time points from 1 to 35 days post fracture for histomorphometry (percent of cartilage and bone), radiographic analysis (total callus volume, callus index, and relative bone mineral content).

**Results:**

The elderly mice produced an equal amount of cartilage when compared to young mice (*p* > 0.08). However, by day 21 there was a significantly greater percentage of bone at the fracture site in the young group (mean percentage 50% versus 11%, *p* < 0.001). It was not until day 35 when the elderly group produced a similar amount of bone compared to the young group at 21 days (50% versus 53%, non-significant (ns)). The callus area and callus index on radiographic assessment was not significantly different between young and elderly groups at any time point. Relative bone mineral content was significantly greater in the young group at 14 days (545.7 versus -120.2, *p* < 0.001) and 21 days (888.7 versus 451.0, *p* < 0.001) when compared to the elderly group. It was not until day 35 when the elderly group produced a similar relative bone mineral content as the young group at 21 days (888.7 versus 921.8, ns).

**Conclusions:**

Elderly mice demonstrated a delay in endochondral ossification which was associated with a decreased relative bone mineral content at the fracture site and may help assess these cellular changes in a clinical setting.

## Introduction

The incidence of fractures is greatest in the elderly population [1]. There is currently limited clinical evidence to support that fracture healing is influenced by age in adult patients [2]. Gruber et al. [3] after reviewing the literature suggested that advancing age has a significant impact on skeletal repair, but much of evidence supporting this is derived from rodent fracture models. An epidemiological study demonstrated that the incidence of fracture non-union to peak in elderly females (25–40 per 100,000), which supports the influence of age on fracture healing in humans [4]. Furthermore, it is generally accepted that fractures in children heal at a faster rate; this is thought to be due to a larger subperiosteal haematoma and a thicker periosteum which may contribute to more rapid formation of callus. [5, 6]

Studies reporting fracture healing in rats have demonstrated that the formation of cartilage and bone, and cartilage resorption are delayed in elderly animals [7]. There is further evidence that ossification of the callus is reduced in older animals [8, 9]. Lu et al. [10] in their original study reported age-related changes in fracture healing in mice, demonstrated delayed peak rates of cartilage formation and endochondral ossification in elderly mice. This was later affirmed by Lopas et al. [11], however in contrast they found the size of the callus to be diminished when compared to younger mice. One hypothesis for this difference is due to the inadequate resolution of inflammation at then fracture site secondary to pro-inflammatory status that is associated with aging term “inflamm-aging” [12]. Despite these cellular differences associated with fracture healing and older age, the radiographic appearance of the fracture site has not, to the authors knowledge, been described according to the observed cellular changes. If the radiographic appearance is related to the differing histological appearances of fracture healing this may enable clinical studies to differentiate those patients with normal fracture healing, according to age, and those with delayed or potential to go onto non-union early in their healing phase.

The aim of this study was to assess the cellular age-related changes in fracture repair and relate these to the observed radiographic assessments at differing time points.

## Materials and methods

### Generation of stable tibial fractures

Ethical approval was obtained from the local ethics committee for all animal procedures performed as part of this study. The young (12–14 weeks old) and elderly (18-month-old) Balb/C wild mice were used in this animal experimental model. Sixteen young (control group) and 20 elderly animals were assigned to each age group, with 4 being used to assess fracture healing at different time points up to 21 days in the young group and 35 days in the elderly group (Table 1). The time points for assessment of 1, 7, 14 and 21 days were chosen as there were thought to represent key stages of healing: inflammatory, fibrous callus, chondrogenic and endochondral stages, respectively. [13] In addition, a 35 day assessment was included for the elderly group only to ensure any delay in fracture healing could be assessed. All animals were kept in the same conditions (cage, bedding, temperature, light). Prior to anaesthesia the animal was weighed, and the mass recorded. Anaesthesia was induced by gas inhalation using Isoflurane at a concentration of 5% (in 10 L of oxygen) and was used for maintenance of anaesthesia at a dose of 1–2%. A satisfactory level of anaesthesia was confirmed by loss of corneal reflex and withdrawal response and by monitoring of the pattern and depth of respiration. Closed fractures of the tibia were performed using a three-point bending technique. Fractures were then stabilised using a plaster cast. Tibias were collected at the defined time points using carbon monoxide. They were immediately immersed in 4% formaldehyde in PBS at pH 7.2–7.4 and left for 48 h and samples were then completely decalcified after 3 weeks at 37 °C with weekly changes of EDTA, after which they were embedded in paraffin. Sagittal Sects. (5 µm) were cut on a standard microtome (Shandon, Thermo Fisher Scientific™, Waltham, MA) and mounted on “Superfrost plus” slides (BDH biosciences™, Poole UK) after floating on deionised water at 40 °C to remove ridges.

**Table 1:** Number of animal euthanized at the different time points according to group

Group	Time post-fracture (days)				
	1	7	14	21	35
Young (*n*)	4	4	4	4	–
Elderly (*n*)	4	3*	4	4	4

*One mouse died due to post-surgical mortality before 7 days and was removed from the study

### Histological analysis

The general morphology of the healing fractures was assessed using three slides from each specimen at each time point was stained with haematoxylin and eosin (H&E). Sections were taken 50 µm apart to control for variability throughout the specimen. A general descriptive assessment of morphology was made using light microscopy at magnification from 40 × to 400 ×, and a. Formal quantification of tissue types present at the fracture site at day 7, 14, 21, and 35 (elderly group only) was performed by photographing images of the fracture callus at a magnification of 100 × using the microscope camera (Nikon DXM 1200) and image grabber software. A 10 × 10 grid was placed over the image on the computer screen (Adobe Photoshop Elements 3.0 ™). This applied 100 cross points to a field of view each corresponding to an area of 0.03mm^2^. The fracture site was defined as beginning where the periosteum became elevated to where it returned to cortical contact beyond the fracture. The fracture site was examined systematically and the number of cross points that overlaid each different tissue type was counted. Tissue types were recorded as: newly formed bone, cartilage, and undifferentiated tissue. The results were expressed as the number of points for each tissue type at the fracture site over the total number of points mapped (100) and the results were expressed as a percentage. The results from the three sections were averaged to provide a mean score for each specimen [14].

### Histomorphometric analysis

Histomorphometry was used to quantify the formation of cartilage and new bone at the fracture site. Three sections from each specimen at each time point were stained with safranin-O (to quantify cartilage which is stained red) and Goldner’s modified mason’s trichrome (was used to quantify bone formation which is stained blue) [15]. Three sections for each stain were taken at 50 µm apart to control for variability throughout the specimen. Two images were taken from each section randomly throughout the callus area, with an ultimate yield of 6 images per fracture at each time point. This therefore gave a uniform distribution of the tissue present and was equal to the minimal nominal value required to reach statistically valid measurements representative of the whole callus tissue [16]. The images were digitised using the microscope camera (Nikon DXM 1200) and image grabber software and transferred into the histomorphometry software package (Bioquant-USA). This was calibrated for 200 × magnification to allow real measurements of area in µm^2^. The number of pixels comprising each tissue component was used to estimate the area and this was determined by selecting the pixels using the lasso tool. Cartilage size was determined by selecting pixels stained by safranin-O. Size of newly formed mineralised bone was determined by selecting pixels stained blue after trichrome staining. The standard total field area of each image was known and constant, and hence the proportion of the callus comprising cartilage and bone was determined and expressed as a percentage of the total area for each time point. [10, 15, 16].

### Radiographic analysis

Radiographic analysis was obtained at time of schedule euthanasia. The animals were placed on a tabletop X-ray unit (Faxitron X-ray Corporation, IL, USA) and radiographed using an exposure of 60 kV output for 0.6 s and a focal distance of 72 cm. Radiographs were taken in both antero-posterior (AP) and lateral planes, and the planes were controlled in each animal using a specifically designed jig (which also controlled for magnification). The animal was positioned centrally using laser crosshairs for guidance. The radiographs produced from this were scanned using a high-resolution scanner (UMAX™ powerlook 2100XL, 500dpi) specially designed to scan radiographs. Because each radiograph had been taken with a steel number (all of the same density and completely radio-opaque) to label the images they were standardised by setting the grey-level over each steel number to white, within the image analysis software [17]. Digital images were then saved in Tagged Image File Format (TIFF) and analysed using Image J™ image analysis software.

The callus area was measured using pixilation: the marquee tool was used to delineate the callus circumferentially in the 2-dimensional plane. The number of pixels contained within the selected area was then recorded in squared inches (as the scanner scanned in dpi) on the lateral radiograph was recorded, which has previously been described [14]. The AP radiograph was not used as the fibula image overlying the tibia would have confounded the results [17].

The callus index is the maximum transverse width of the callus divided by the width of the bone at the fracture site. This was determined using the image software, for both AP (medial–lateral) and lateral (antero-posterior) radiographs, using pixilation as previously descibed [18]. This method corrects for any initial differences in bone size between animals.

Relative bone mineral content was assessed using pixel density across the fracture gap. The mean pixel density of two areas in the fracture gap was compared to two adjacent areas of uninjured bone using Image J™ software at each time point. Initially there is less bone at the fracture gap than in the adjacent areas, however with healing callus is laid down and the pixel density becomes relatively greater than in the uninjured area. The mean density value within the fracture gap is subtracted from the mean density value in the normal bone and these values represent ‘relative bone mineral content’. The intra and inter-observer variability of pixel density analysis has been demonstrated to be highly reproducible [19].

### Statistics

Statistical analysis was performed using Statistical Package for Social Sciences version 17.0 (SPSS Inc., Chicago, IL, USA). Non-parametric tests were used to assess continuous variables for significant differences between groups. A Mann Whitney U test or a Kruskal–Wallis test were used to compare scale variables between groups and change within a group over time, respectively. A *p* value of ≤ 0.05 determined statistical significance. A power calculation was performed and a four animals per time point (7, 14 and 21 days) in each group would achieve 80% power using a 0.05 alpha value.

## Results

### Histology analysis

**Fig. 1:**
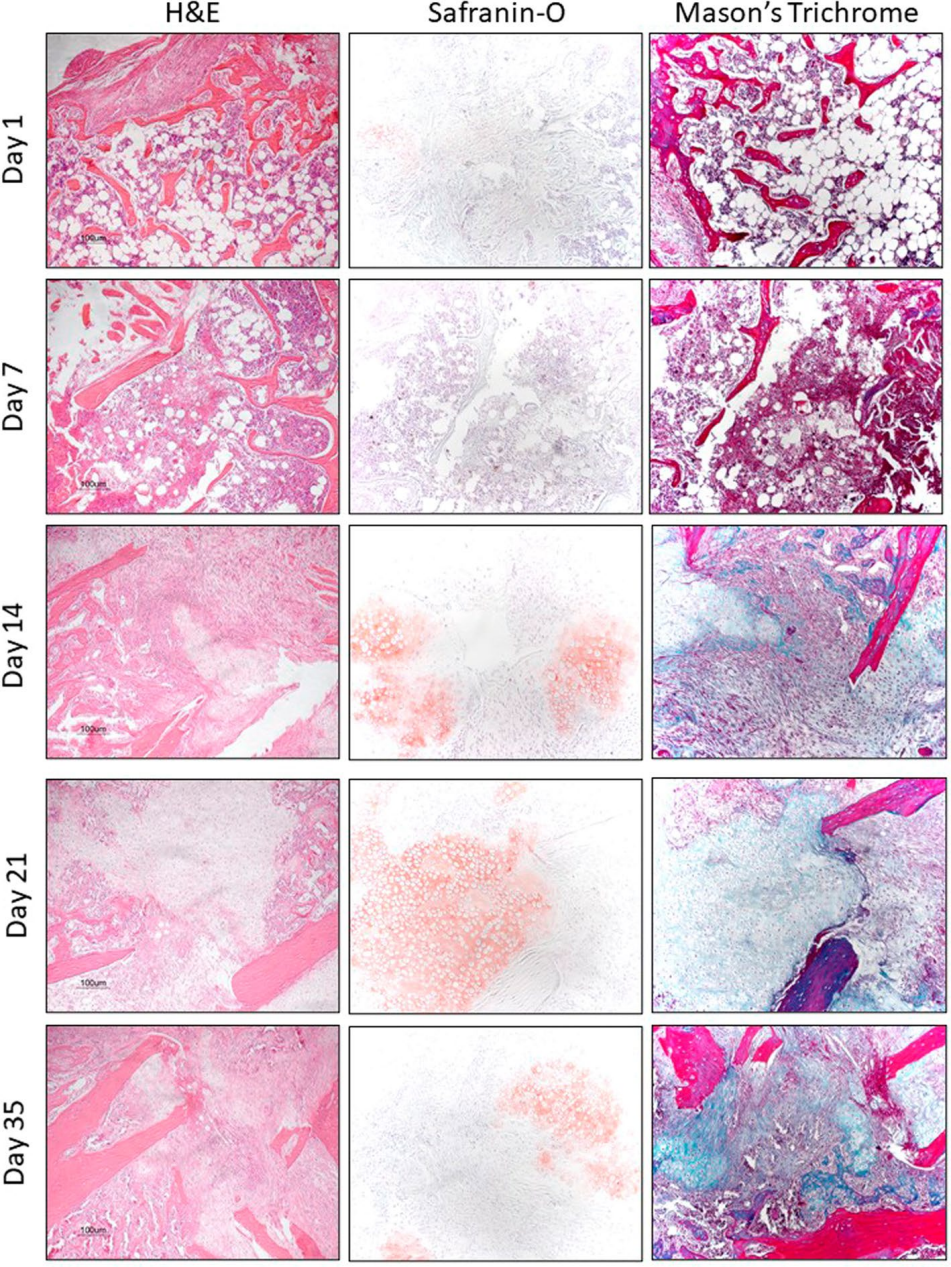
Haematoxylin and eosin, safranin-O (cartilage stained red) and Goldner’s modified mason’s trichrome (bone stained blue) stains at the fracture site of the elderly mice group according to time from fracture

**Fig. 2:**
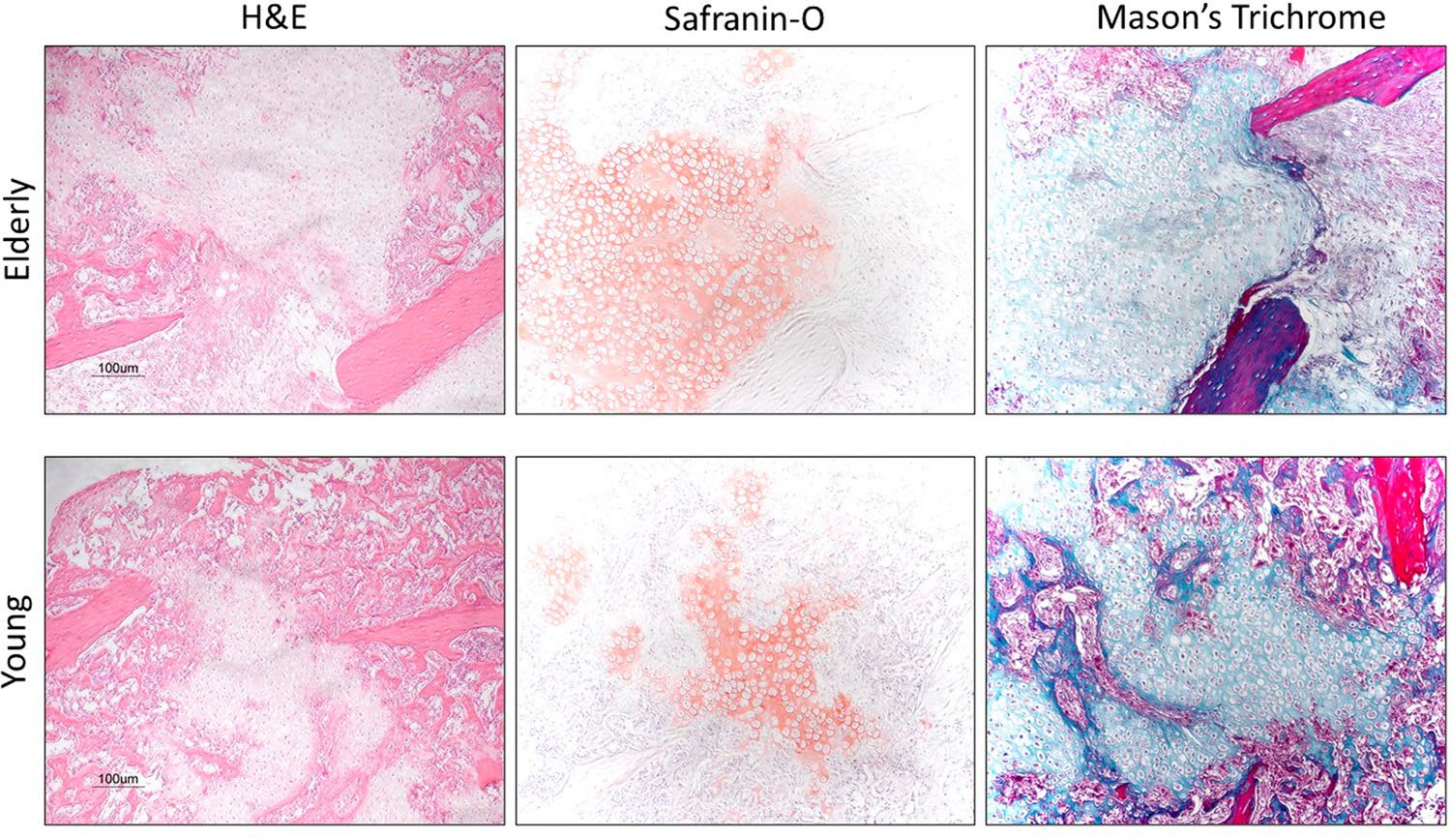
Haematoxylin and eosin, safranin-O (cartilage stained red) and Goldner’s modified mason’s trichrome (bone stained blue) stains at the fracture site of the elderly mice group and the young mice group 21 days from fracture

There were no discernible differences between groups at day 1 post fracture, which was composed mainly of haematoma and an influx of polymorphonuclear leukocytes and monocytes (Fig. 1). There were still no apparent differences between the groups at day 7 post fracture with an abundance of undifferentiated tissue and limited cartilage formation. Again, at day 14 there was no obvious difference between the groups with callus now fully bridging the fracture gap and ossification commencing in some areas. By day 21 there was an obvious difference between the groups, with a greater amount of ossification being observed in the young group (Fig. 2), which was not recognised until day 35 in the elderly group. Quantification of the cartilage and bone formation in the fracture site demonstrated no significant difference between the groups at days 7 or 14, but by day 21 there was significantly more bone and a trend towards less cartilage being observed in the young group (Table 2). There were no significant differences observed between the amount of cartilage or bone between the young group at 21 days and elderly group at 35 days. There was significant increase in the proportion of cartilage and bone with the callus for both groups relative to that observed at day 7 (Table 2).

**Table 2:** Quantification of cartilage and bone formation within the fracture site as a percentage of the callus at the different time points according to group

Day	Cartilage (%)			Bone (%)		
	Young	Elderly	*p* value*	Young	Elderly	*p* value*
7	12 (7)	13 (8)	ns	0	0	ns
14	56 (8)	51 (9)	ns	7 (3)	4 (3)	ns
21	46 (7)	53 (8)	0.08	50 (9)	11 (4)	< 0.001
35	–	46 (8)	ns^α^	–	53 (8)	ns^α^
*p* value**	< 0.001	< 0.001		< 0.001	< 0.001	

*Mann Whitney *U*

**Kruskal–Wallis

^α^This *p* value represents the comparison of the young group at 21 days with the elderly group at 35 days

### Histomorphometry analysis

There were small pockets of cartilage defined by the Safranin-O staining at 7 day in both groups, which increased significantly by day 14 and accounted for approximately 60% of the callus composition with no significant difference between the groups at either time point (Table 3). At day 21 the proportion of cartilage observed in the callus was significantly less in the young group compared to the elderly group, however this difference was not observed at 35 days in the elderly group compared to the 21 day proportion of cartilage in the young group (Table 3). There was no bone formation within the callus site at 7 days for either group defined by Mason’s trichrome (Fig. 1). At 14 days there were small areas of ossification which were significantly more frequent in the young group account for a greater proportion of the callus (Table 3). The young group had a greater rate of ossification, and by day 21 more than 50% of the callus had ossified which was significantly greater than the elderly group (Table 3). However, by day 35 the elderly group there was no significant difference in the proportion of bone compared to the young group at 21 days. These results suggest that the rate of cartilage formation during the first 14 days was not affected by age, but in contrast the rate of ossification of the soft callus would seem to be delayed by up to 14 days in the elderly group. These findings are consistent with the observed H&E histology.

**Table 3:** Quantification of the proportion of cartilage using Safranin-O staining and bone using Mason’s trichrome within the callus at the different time points according to group

Day	Cartilage (%)			Bone (%)		
	Young	Elderly	*p* value*	Young	Elderly	*p* value*
7	7.0 (1.5)	6.2 (2.3)	ns	0	0	ns
14	60.9 (2.5)	56.7 (3.6)	ns	15.0 (1.5)	5.6 (1.8)	0.004
21	39.7 (1.9)	58.1 (4.2)	< 0.001	53.2 (3.2)	14.4 (4.0)	< 0.001
35	–	37.2 (3.8)	ns^α^	–	54.7 (4.1)	ns^α^
*p* value**	< 0.001	< 0.001		< 0.001	< 0.001	

*Mann Whitney *U*

**Kruskal–Wallis

^α^This *p* value represents the comparison of the young group at 21 days with the elderly group at 35 days

### Radiographic analysis

**Fig. 3:**
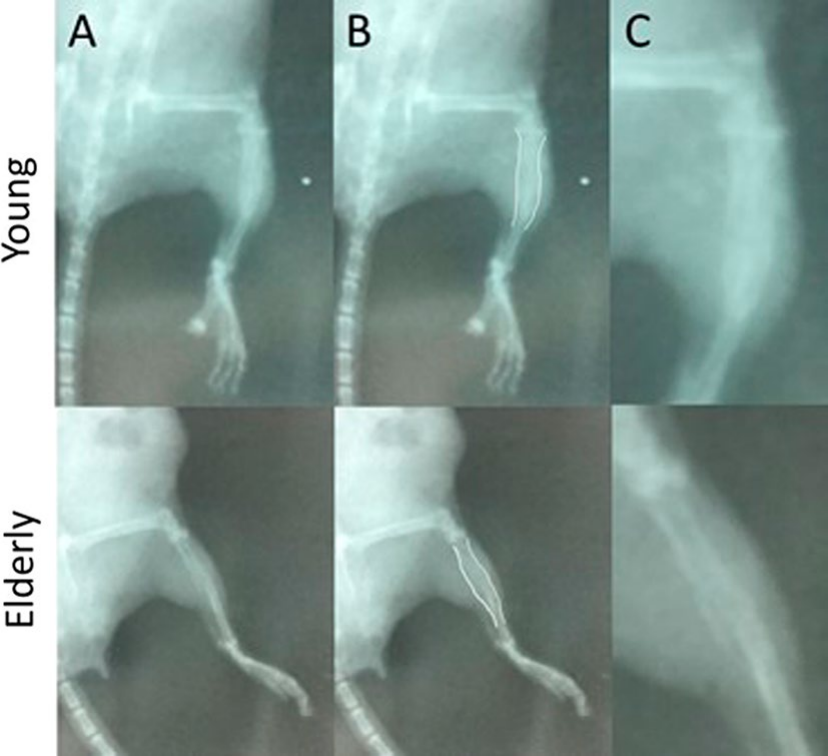
Radiographs of the tibial fracture site (**A**) in an elderly and a young mice at 21 days from injury. The callus area has been demarcated in white (**B**) and the fracture site has been magnified (**C**) to illustrate the difference in density at the fracture site

The callus area increased significantly for all time points assessed relative to the callus area at day 7 for both groups. The callus area at day 35 in the elderly group did not increase relative to that measured on day 21, suggesting the peak callus area had been achieved. There was however, no significant difference in the callus area at any time point between the two groups (Fig. 3), and if there were a trend the elderly group had a marginally larger area (Table 4).

**Table 4:** Callus area measured on lateral radiographs at the different time points according to group

Day	Callus area (inch^2^)		
	Young	Elderly	*p* value*
7	0.007 (0.002)	0.008 (0.002)	ns
14	0.014 (0.002)	0.016 (0.003)	ns
21	0.022 (0.003)	0.023 (0.003)	ns
35	–	0.025 (0.004)	ns^α^
*p* value**	< 0.001	< 0.001	

*Mann Whitney *U*

**Kruskal–Wallis

^α^This *p* value represents the comparison of the young group at 21 days with the elderly group at 35 days

There was a similar trend observed for the callus index as demonstrated with the callus area. There was a significant increase in the callus ratio, on both AP and lateral radiographs, at day 21 compared to day 7 for both groups and for day 35 compared to day 7 for the elderly group (Table 5). Again, the callus index at day 35, on both AP and lateral radiographs, in the elderly group did not increase relative to that measured on day 21, suggesting the peak callus index had been achieved. There were however, no significant differences in the callus index at any time point between the two groups, or on comparison of the day 35 index in the elderly with the day 21 index in the young group (Table 5).

**Table 5:** Callus index (ratio) measured on AP and lateral radiographs at the different time points according to group

Day	AP radiograph			Lateral radiograph		
	Young	Elderly	*p* value*	Young	Elderly	*p* value*
7	1.45 (0.08)	1.51 (0.12)	ns	1.39 (0.11)	1.32 (0.19)	ns
14	1.50 (0.09)	1.61 (0.11)	ns	1.67 (0.11)	1.80 (0.21)	ns
21	2.86 (0.47)	2.84 (0.38)	ns	2.49 (0.31)	2.51 (0.36)	ns
35	–	2.91 (0.41)	ns^α^	–	2.55 (0.42)	ns^α^
*p* value**	< 0.001	< 0.001		< 0.001	< 0.001	

*Mann Whitney *U*

**Kruskal–Wallis

^α^This *p* value represents the comparison of the young group at 21 days with the elderly group at 35 days

The relative bone mineral content significantly increased for all time points relative to that measured on day 7 for both groups (Table 6). There were no significant differences between the groups for relative bone mineral content at day 7, but by days 14 and 21 the young group had a significantly greater relative bone mineral content compared to the elderly group (Fig. 3 and Table 6). However, by day 35 the elderly group had a relative bone mineral density similar to that observed in the young group at day 21, with no significant difference.

**Table 6:** Relative bone mineral content measured at the fracture site for the different time points assessed according to group

Day	Relative bone mineral content		
	Young	Elderly	*p* value *
7	− 961.5 (72.5)	− 910.3 (63.8)	ns
14	545.7 (53.9)	− 120.2 (72.9)	< 0.001
21	888.7 (57.7)	451.0 (83.9)	< 0.001
35	–	921.8 (61.9)	ns^α^
*p* value**	< 0.001	< 0.001	

*Mann Whitney *U*

**Kruskal–Wallis

^α^This *p* value represents the comparison of the young group at 21 days with the elderly group

## Discussion

This study has confirmed the cellular age-related changes associated with fracture repair and has demonstrated these changes to be evident on radiographic analysis. The rate of cartilage formation at the fracture site was not influenced by age, which is supported by histological evidence and on Safranin-O staining that demonstrated no difference between young and elderly groups. There was also no difference in the radiographic assessment at any time point in the callus area or callus index according to age. However, the rate of ossification was influenced by age, analysis of both histological sections and Mason’s Trichrome staining demonstrated a significantly greater proportion of new bone formation the young group at days 14 and 21 compared to the elderly group. The rate of ossification would seem to be delayed by approximately 14 days, with the elderly group requiring 35 days to produce a similar proportion of new bone formation as observed at day 21 in the young group. This was also evident on the radiographic analysis which demonstrated a greater relative bone mineral content, being a marker of ossification, in the younger group at days 14 and 21. The elderly attained the same relative bone mineral content as the young group, but this again was not evident until day 35 in contrast to day 21 in the young group.

Meyer et al. [8] demonstrated that normal biomechanics of the femurs in younger rats was restored within 4 weeks of fracture, whereas older rats the normal biomechanical strength was not attained until 26 weeks. Using a rabbit model, O'Driscoll et al. [20] harvested the periosteum tibias of rabbits aged 2 weeks to 2 years. They demonstrated that chondrogenesis declined significantly with age and was associated with a thinner periosteum. At the cellular level there is decreased: responsiveness of mesenchymal progenitor cells to signalling molecules, number and division capacity, angiogenesis, osteoinductive activity, and local and systemic levels of signalling molecules with increased age [21, 22]. This may explain some of the histological and radiographical differences observed in the current study. More recently Clark et al [23] have shown that age related changes in the macrophages infiltrating the fracture site were detrimental to healing, which they suggest is due to dysregulation of pro-inflammatory genes. Xiao et al. [24] showed that periosteal stems cell that decrease in number with aging are critical to bone regeneration, that may be reversible in aged mice with appropriate biochemical signal stimulation. Liu et al. [25] have also shown Wnt/β-catenin signalling to promote more effective fracture healing in aged mice by inducing cell differentiation and angiogenesis. Therefore, manipulation of the inflammatory response to healing and signalling pathways in older animals/patients may influence the rate of fracture healing.

Lu et al. [10] using a murine model demonstrated delayed peak rates of cartilage formation and endochondral ossification in elderly relative to younger mice after induced diaphyseal femoral fractures. The current study supports these findings, with equal amounts callus formation but a significant delay in the rate of endochondral ossification. Lopas et al. [11] also found a delay in the rate of ossification, but in contrast they described a significant reduction in the size of soft callus in the elderly group. The reason for the difference in size of callus formation between Lu et al. [10] and the current study compared with Lopas et al. [11] is not clear. Potential factors could be that Lopas et al. [11] used older mice (25 month) and did not stabilize their fractures post injury, whereas the current study employed cast stabilization which has been shown to influence the size of callus formation and rate remodeling. What is affirmed by all studies is that the rate of endochondral ossification is delayed in elderly mice. More recent studies by Lu et al. [10] attributed these changes in fracture healing to the differing rate of angiogenesis which decreases in older mice [26], which may be related to the level of oxygenation at the fracture site [27].

There are numerous radiographic tools available to assess the quality of fracture healing. Callus ratio is one such tool, which has been demonstrated to be an indicator of healing when assessed over time. However, the current study did not identify a difference in callus ratio according of age, as there was no significant difference in the size of the callus at any time point. The radiographic union score for tibial (RUST) fractures is a novel fracture assessment tool that has been shown to have reliable intraobserver and interobserver agreement compared to other tools [28, 29]. This score, although not assessed in the current study, may be influenced by age. The RUST assesses for bridging callus between the bone ends at the fracture site, which if the rate of ossification is delayed as demonstrated in this study may take longer for the bridging soft callus to calcify and therefore may not be observed radiographically. The current study used bone mineral content measured at the fracture which was significantly different between the groups which seemed to relate to the degree of endochondral ossification. This measure could be used in conjunction with the RUST to assess the degree of ossification of the bridging callus to ensure biomechanical stability of the fracture site before weight bearing is commenced. Further work and quantification studies would be needed to assess what measure of bone mineral content relates to the amount of endochondral ossification and when biomechanical stability was achieved.

A major limitation of this study, and other studies investigating age related changes of fracture healing, is using a diaphyseal model despite most elderly fractures occurring in the metaphysis [1]. Hence the findings may not be clinically relevant when managing elderly patient fractures. A review identified this as a major limitation of the current evidence and suggested future animal models include three criteria: metaphyseal induced fracture site, complete discontinuity with metal implants for stabilization, in ovariectomized aged rodents [30]. The biomechanical properties of the fracture (stiffness and strength) were not assessed, which may have demonstrated a difference between the groups due to the observed delay in calcification in the elderly mice. Also, the current study did not assess the molecular mechanisms influencing the observed cellular changes and differences according to age, an understanding of which may help future clinical practice to influence the rate of fracture healing.

The delay in endochondral ossification associated with aging can be graded on radiographs which may help assess these cellular changes in a clinical setting. This could be used with or without other assessment tools to evaluate factors influencing the fracture healing in elderly patients.
